# Prevalence of high-grade squamous intraepithelial lesions and cervical cancer among patients with unsatisfactory colposcopic examination, without visible lesion

**DOI:** 10.1590/S1516-31802009000500004

**Published:** 2010-02-03

**Authors:** Fernanda Rangel da Veiga, Fábio Bastos Russomano, Maria José de Camargo, Aparecida Cristina Sampaio Monteiro, Aparecida Tristão, Gabriela Villar e Silva

**Affiliations:** 1 MD, MSc. Gynecologist, Instituto Fernandes Figueira (IFF), Fundação Oswaldo Cruz (Fiocruz), Flamengo, Rio de Janeiro, Brazil.; 2 MD, MSc, PhD. Gynecologist, Department of Gynecology and Obstetrics, Colposcopy Clinic, Instituto Fernandes Figueira (IFF), Fundação Oswaldo Cruz (Fiocruz), Rio de Janeiro, Brazil.; 3 MD, MSc, PhD. Gynecologist, Instituto Fernandes Figueira (IFF), Fundação Oswaldo Cruz (Fiocruz), Flamengo, Rio de Janeiro, Brazil.; 4 MD, MSc. Gynecologist, Department of Gynecology and Obstetrics, Colposcopy Clinic, Instituto Fernandes Figueira (IFF), Fundação Oswaldo Cruz (Fiocruz), Rio de Janeiro, Brazil.; 5 MD, MSc. Pathologist, Universidade Federal Fluminense (UFF), Niterói, Rio de Janeiro, Brazil.; 6 Medical student, Universidade do Grande Rio (Unigranrio), Rio de Janeiro, Brazil.

**Keywords:** Cervical intraepithelial neoplasia, Carcinoma, Colposcopy, Conization, Public Health, Neoplasia intra-epitelial cervical, Carcinoma, Colposcopia, Conização, Saúde Pública

## Abstract

**CONTEXT AND OBJECTIVE:**

Cervical cancer is a serious public health problem in Brazil. For patients with unsatisfactory colposcopic examinations without visible lesions, but with cervical cytological tests suggesting high-grade squamous intraepithelial lesion (HSIL), the national recommendation is to repeat cervical cytological tests after three months. Our aim was to assess the prevalence of HSIL and cancer among patients with initial cervical cytological tests suggestive of HSIL but with unsatisfactory colposcopic examinations without visible lesions, in order to contribute towards the discussion regarding a more effective clinical approach that might diminish the likelihood of patient abandonment of follow-up before appropriate diagnosis and treatment.

**DESIGN AND SETTING:**

Cross-sectional study in Colposcopy Clinic of IFF/Fiocruz.

**METHOD:**

Patients admitted between December 1989 and April 2007 with cytological diagnoses of HSIL but with unsatisfactory colposcopic examinations without visible lesions underwent cervical cone biopsy.

**RESULTS:**

Sixty-five such patients were included, comprising 33.8% with HSIL and 4.6% with cancer, confirmed histologically. The other patients presented low-grade squamous intraepithelial lesion (26.1%), glandular dysplasia (1.5%) and absence of disease (33.8%).

**CONCLUSION:**

The observed prevalence of cancer and HSIL does not seem to be enough to justify immediate referral for cone biopsies to investigate the cervical canal in these cases. The findings suggest that the recommendation of repeated cytological tests following an initial one with HSIL, among patients with unsatisfactory colposcopic examinations without visible lesions, is appropriate in our setting. Efforts are needed to ensure adherence to follow-up protocols in order to reduce the chances of losses.

## RESUMO

**INTRODUÇÃO:**

O câncer de colo uterino é um grave problema de saúde pública no Brasil. Em pacientes com colpocitologias sugestivas de lesão intra-epitelial escamosa de alto grau (HSIL) e colposcopia insatisfatória sem lesão visível, a recomendação nacional é repetir a colpocitologia após três meses. Nosso objetivo foi medir a prevalência de HSIL e câncer em pacientes com a primeira colpocitologia sugestiva de HSIL e colposcopia insatisfatória sem lesão visível, no intuito de contribuir para a discussão sobre uma conduta clínica mais efetiva e que diminua a probabilidade de perdas de acompanhamento antes do diagnóstico e tratamento adequados.

**TIPO DE ESTUDO E LOCAL:**

Estudo transversal no Ambulatório de Colposcopia do IFF/Fiocruz.

**MÉTODO:**

Pacientes recebidas no período de dezembro de 1989 a abril de 2007 com diagnóstico citológico de HSIL sem lesão visível em colposcopias insatisfatórias foram submetidas a conização do colo uterino.

**RESULTADOS:**

Foram incluídas 65 pacientes na situação descrita e encontrados 33,8% de HSIL e 4,6% de câncer confirmados histologicamente. Os demais casos apresentaram lesão intra-epitelial escamosa de baixo grau (26,1%), displasia glandular (1,5%) e ausência de doença (33,8%).

**CONCLUSÃO:**

A prevalência de HSIL ou câncer encontrada não parece suficiente para defender a conduta de encaminhar as pacientes de imediato para conização a fim de investigar o canal cervical. Os achados sugerem que a recomendação de repetir a citologia após uma primeira com HSIL sem lesão visível e colposcopia insatisfatória é apropriada no nosso cenário. Devem ser implementados esforços para adesão às recomendações de acompanhamento e reduzir a chance de perdas.

## INTRODUCTION

Despite the existence of a public program to prevent cervical cancer, this disease is the second most frequent type of neoplasia among Brazilian women.^1^

In 1997, the Programa Nacional de Controle do Cancer do Colo do Útero e da Mama (National Program for Cervical and Breast Cancer Control) was established. It recommended standard procedures for the management of cervical cytological abnormalities among Brazilian women receiving publicly funded healthcare. One of these recommendations stated that women with a cytological diagnosis of high-grade squamous intraepithelial lesion (HSIL) without visible lesions seen on colposcopic examination, and a transformation zone that is incompletely seen need to undergo the cytological test again in three months’ time if review of the slides is not possible or the review does not change the previous diagnosis. If this diagnosis is sustained in the second sample, these women must undergo an excisional procedure.^2^

However, in the Colposcopy Clinic of Instituto Fernandes Figueira, which is a healthcare and medical research and teaching unit of Fundação Oswaldo Cruz (IFF/Fiocruz), it was decided to proceed with endocervical investigation in these cases, by performing cervical conization. The intention was to avoid possible losses from follow-up among women with true HSIL who were unable to attend follow-up medical appointments. This strategy is stated to be acceptable in the American consensus.^3^

It is important to note that the Brazilian national recommendation is based on a consensus among specialists, and it is not based on any medical evidence. The purpose of this paper was to contribute towards this discussion regarding the national program.

## OBJECTIVE

Our objective was to measure the prevalence of HSIL and cervical cancer among women with a cytological diagnosis of HSIL who were referred to a colposcopy clinic, at which their colposcopic examination did not present any visible lesion but was unsatisfactory.

## METHOD

This was a cross-sectional study using retrospective data on patients referred from primary healthcare units or private consultation offices. Most of the patients lived in the city of Rio de Janeiro or other municipalities in the state of Rio de Janeiro. Data were colleted from a database and confirmed using the original patients’ charts.

The criterion for patient inclusion was that they should be women with cervical cytological tests suggestive of HSIL who had been referred to the colposcopy clinic of IFF/Fiocruz colposcopy as part of an existing referral system for diagnosing and treating the precursors of cervical cancer. They showed no visible lesion under colposcopy during an unsatisfactory colposcopic examination and had already undergone cervical conization to avoid possible losses from follow-up. The colposcopic examination was considered unsatisfactory when the transformation zone was incompletely seen, even after maneuvers to display the squamous-columnar junction (SCJ). Conjugated estrogens (0.625mg p.o./day) was prescribed for seven to ten days before a second colposcopic examination. A new colposcopic examination was also performed when the first one was limited by colpitis or bleeding. In these cases, the examination was repeated as soon as possible, after the treatment for the condition that impaired the first one. The colposcopic examinations were always performed by senior colposcopists or under their supervision.

Cases with inconclusive histological reports due to thermal artifacts were excluded from the analysis.

The prevalence of HSIL and cancer was calculated using the Epi-Info software version 6.04, along with the prevalence of any other diagnoses found in the cone biopsies, with the 95% confidence interval (CI).

This study was approved by the Ethics Committee of Instituto Fernandes Figueira, Fiocruz, in May 2006. This was a retrospective study, using data from a local database. All cases were included after their treatment had been completed (thus the informed consent form was considered unecessary).

## RESULTS

Sixty-five patients with a cytological diagnosis of HSIL and unsatisfactory colposcopic examinations without visible lesions were included between December 1989 and April 2007. These women were among the patients referred to IFF/Fiocruz for confirmation and treatment of preinvasive cervical disease and for cervical cancer prevention. The age range of the patients included was from 27 to 70 years, with a mean of 46 years and a median of 45 years.

Table 1 shows the distribution of cone biopsy diagnoses. The prevalence of HSIL or cervical cancer was 38.4% (95% CI: 27.2-50.6%).

**Table 1.** Distribution of histological diagnoses of cone biopsy specimens from 65 patients with cytological diagnoses of high-grade squamous intraepithelial lesion (HSIL) and unsatisfactory colposcopic examinations without visible lesions (Instituto Fernandes Figueira, Fundação Oswaldo Cruz, 1989-2007)

Histological diagnosis	Prevalence n (%)	95% confidence interval
HSIL	22 (33.8%)	23.1-45.9%
Cervical cancer	3 (4.6%)	1.1-12.0%
Glandular dysplasia	1 (1.5%)	0.07-7.35%
LSIL/HPV	17 (26.3%)	16.5-37.8%
Cervicitis/normal	22 (33.8%)	23.1-45.9%
Total	65 (100%)	

LSIL = low-grade squamous intraepithelial lesion; HPV = human papillomavirus.

## DISCUSSION

The results from this study may be applicable to women who use the public healthcare system and private consultation offices in the city of Rio de Janeiro, and to other similar populations.

The prevalence of HSIL and cancer found in this sample was less than prevalences found by other authors. Massad et al.^4^ found a prevalence of HSIL and cervical cancer of 54% in a similar population in which 78 patients with cytological diagnoses of HSIL and unsatisfactory colposcopic examinations underwent electrosurgical cone biopsies. However, these authors did not declare whether there were any visible lesions, which leaves us inclined to believe that they might have included women both with and without visible lesions seen during colposcopy. Thus, the presence of patients with visible lesions among their sample may have increased the prevalence of histological lesions.

A study by Lapin et al.^5^ that was developed in Campinas, Brazil, and was designed to test the diagnostic performance of cervical cytological tests found that the prevalence of histologically diagnosed HSIL was 49.5%, among 123 patients referred with a cytological diagnosis of HSIL. However, only 9.7% of these women had unsatisfactory colposcopic examinations. It was not reported how many of them presented visible lesions in the colposcopic examination. In this group of patients, only 53.6% continued with this cytological diagnosis in a second examination that was performed at the referral center. Among these persistent cases, 71.2% showed histologically diagnosed HSIL. Again, there were no reports of visible lesions. This greater prevalence could be due to the repeated cytological diagnosis of HSIL.

Andersen et al.^6^ determined the prevalence of histologically diagnosed HSIL among patients with a cytological diagnosis in Denmark. They included 296 women and took the final diagnosis to be the one obtained by means of cone biopsy. All of the patients underwent colposcopy, but these data was not analyzed because the authors considered them inaccurate. The sample characteristics were not reported and we cannot state that their sample was similar to the one in the present study. They found that 81% of the diagnoses were histologically HSIL and 6% were cervical cancer. Since the colposcopy findings were not analyzed in their study, we may infer that they included patients both with and without visible lesions seen during colposcopy and both satisfactory and unsatisfactory colposcopic examinations. These facts might explain the high prevalence found.

In another study performed in a colposcopy clinic in Chicago, Massad et al.^7^ investigated 362 women with cytological diagnoses of HSIL, among whom 48% showed a histological diagnosis of HSIL and 6% of cervical cancer. All of the patients underwent colposcopic examination and, for 26% of them, it was unsatisfactory. However, these data was not taken into account in the abovementioned prevalence. The final diagnosis was obtained by means of cone biopsy or hysterectomy. The higher prevalence than among the present study can also be explained by the inclusion of patients with satisfactory colposcopy and with visible lesions seen during colposcopic examination.

From a literature search, in the PubMed and Literatura Latino-Americana e do Caribe em Ciências da Saúde (Lilacs) databases, no other articles reporting on the prevalence of HSIL and cervical cancer in situations of cytological tests suggesting HSIL but without visible lesions seen during an unsatisfactory colposcopic examination were found. Most studies that were in some way related to this topic reported on the prevalence of these diseases in any situation, i.e. both satisfactory and unsatisfactory colposcopic examination and with or without a visible lesion, in their estimates of cytological diagnostic performance.

It has been argued that the prevalence of HSIL and cancer vary according to age. A population study in the United States showed that the proportion of cytological diagnoses of HSIL was higher in the age range from 20 to 29 years (0.6%, n = 44,052).^8^ Another study, performed in India, had a mean age of 37.7 years for the cytological diagnosis of HSIL and 51.8 years for cervical cancer.^9^ This factor can be considered in deciding which strategy can best fit each patient.

Given the usual losses from follow-up among patients using the public healthcare system, the indication of cone biopsy in the colposcopy clinic of IFF/Fiocruz provides a diagnostic and treatment opportunity for a significant number of patients who have true HSIL or cancer. On the other hand, a high proportion of these women underwent an unnecessary surgical procedure. Among these women, we found low-grade intraepithelial lesions (cervical intraepithelial lesion grade 1 or human papillomavirus, HPV, infection) or no lesions at all. Although we used electrosurgical techniques under local anesthesia, and the hospital stay was short, we believe that these procedures should be indicated more conservatively within our setting. Another factor is that the estimated progression rate from HSIL to cancer was 1.44% over a two-year period,^10^ thus indicating that there was virtually no progression over a short period of time. Taking all these data together, we consider that repeating the cervical cytology in three months’ time is useful in our setting, as recommended by the national program for cervical cancer control.^2^ However, women in this situation should be advised to adhere to the follow-up protocol, in order to avoid the loss of the opportunity for cancer prevention.

## CONCLUSION

The low prevalence of HSIL found does not support systematic indication of cone biopsy for women solely presenting cytological diagnoses of HSIL, with unsatisfactory colposcopic examinations that did not show any visible lesions. The findings suggest that the recommendation of repeated cytological tests following an initial one with HSIL, among patients with unsatisfactory colposcopic examinations without visible lesions, is appropriate in our setting. Efforts are needed to ensure adherence to follow-up protocols in order to reduce the chances of losses.

Considering the limitations of observational studies, better conclusions would be drawn from a randomized clinical trial addressing this issue.
